# Is negative urine culture necessary for PCNL safety? Experience from a large-volume stone center

**DOI:** 10.3389/fsurg.2025.1571963

**Published:** 2025-05-30

**Authors:** Xiao Bo, Xue Zeng, Gang Zhang, Chaoyue Ji, Song Jin, Wenjie Bai, Yuzhe Tang, Bixiao Wang, Jianxing Li

**Affiliations:** Department of Urology, Beijing Tsinghua Changgung Hospital, School of Clinical Medicine, Tsinghua Medicine, Tsinghua University, Beijing, China

**Keywords:** PCNL, urine culture, safety, complication, antibiotics

## Abstract

**Objectives:**

To present our large single-center experience in managing patients with positive urine cultures and kidney stones with total ultrasound-guided percutaneous nephrolithotomy (PNL) and to redefine the role of urine culture in modifying these patients’ treatment plans.

**Patients and methods:**

We retrospectively reviewed the charts of patients who had undergone PNL in our department from January 2016 to December 2020 and identified 422 eligible patients. These patients were allocated to two groups according to pre-operative urine culture results: negative (Group 1, *n* = 278) and positive (Group 2, *n* = 144). All procedures were ultrasound-guided. Standard access was achieved in all patients. Relevant patient characteristics, operative variables, and postoperative data were collected and analyzed, focusing on infection-related data, particularly sepsis.

**Results:**

Successful renal access and stone fragmentation were achieved in all patients. At least one standard (24F) tract was established and a negative suction system introduced in every case. *Escherichia coli* was the most common bacterium in positive urine culture patients. Preoperative serum creatinine differed significantly between Groups 1 and 2 (1.2 ± 0.2 mg/dl vs. 2.0 ± 0.7 mg/dl, *p* = 0.02). Durations of surgery (79.2 ± 22.2 min) and post-operative hospitalization (7.6 ± 2.1 days) were longer in Group 2 than in Group 1 (58.2 ± 17.2 min) and (5.6 ± 1.1 days), respectively. Group 1 required fewer renal accesses than did Group 2 (1.1 ± 0.2 vs. 1.7 ± 0.2). The immediate stone-free rate was significantly greater in Group 1 (249; 89.2%) than in Group 2 (108; 75%).

**Conclusions:**

Ultrasound guided PNL with standard access reveals a safe and acceptable results in positive urine culture patients. Preoperative infected urine is not a risk factor for severe septic complications after PNL under controlled conditions.

## Introduction

Large kidney stones are widely managed by percutaneous nephrolithotomy (PNL). Post-operative infectious complications remain the most noteworthy issue among patients undergoing PNL (1). Recent data indicate that up to one-third of such patients develop post-operative complications, fever being the commonest symptom (2). Though very high temperatures are typically transient and can be treated with appropriate antibiotics, 0.9%–9.3% of patients develop life-threatening urosepsis or septic shock (3, 4). Some risk factors for post-operative infectious complications, including female sex and diabetes mellitus, have been identified (5). It has also been suggested that a positive preoperative urine culture is an independent risk factor for post-operative infectious complications (6). Thus, American Urological Association and European Association of Urologists guidelines recommend delaying surgical management until infections have been controlled (7, 8). However, several studies have reported that numerous patients with post-operative infections have negative preoperative urine cultures (9, 10). Researchers have also found that not all patients with persistent bacteriuria develop postoperative fever (11). Because we are a major urolithiasis center, many patients with pyonephrosis or multi-drug resistant (MDR) organisms are referred to us. Urinary tract drainage and adequate antibiotic therapy does not always achieve sterile urine in these patients. Furthermore, we have performed total US-guided standard PNL for more than 15 years in our center. Experience with more than 20,000 cases has proved the safety and efficiency of this procedure in patients with a variety of complex stones. The overall postoperative complication rate is lower in our previous studies than in other reported studies (12–14). In the present study, we retrospectively reviewed our single-center experience with a large cohort with the aim of reporting how we manage these patients with PNL and the outcomes according to preoperative urine culture status.

## Patients and methods

### Patients

The study cohort comprised 422 consecutive patients, data having been obtained from our specialized electronic database (Redcap). The study was approved by the medical ethics committee of our hospital. We included all patients eligible for PNL with the following exceptions: horse-shoe kidney, spinal deformity, pediatric patient, and mini-perc only. Pre-operative midstream urine cultures were collected from all patients,. Positive cultures were defined as microorganism colony number >10^4^ cfu/ml. Oral antibiotics were given for 2–3 days to patients with negative cultures. Empirical antibiotics (cephalosporin/ofloxacin) were IV administered routinely for 2–3 days to those with positive cultures and adjusted according to findings of urine analysis as necessary. The group 1 was defined as patients with bacteria in midstream before procedure after antibiotic therapy. Fever was defined as temperature >38°C. Urosepsis was defined as meeting two or more criteria of quick sepsis-related organ failure assessment: namely respiratory rate >22/min, altered consciousness, and systolic pressure <100 mmHg according to the recent international consensus definitions of sepsis (15). Postoperative complications were assessed according to the Clavien–Dindo grading system.

Routine investigations, such as blood cell count and procalcitonin, were also performed. Radiological examinations, including plain films of the kidney, ureter, and bladder (KUB), ultrasound (US), and non-enhanced or enhanced CT, were used to evaluate the anatomic structure of the collecting system if renal function studies were normal. Stone size was calculated according to the longest size on CT scan. Residual stones were defined as >4 mm stone in post-KUB/CT image after 1-month follow-up. Stone composition was analyzed by infrared spectroscopy. Patient pre- and post-operative characteristics are listed in Table 1.

**Table 1:** Study patients’ characteristics and perioperative data.

Parameters	Group 1 (*n* = 278)	Group 2 (*n* = 144)	*P* value
Age (years), mean ± SD	56.4 ± 13.9	58.2 ± 14.9	0.43
Gender (male/female)	180/98	43/101	<0.001
BMI (kg/m^2^), mean ± SD	24.5 ± 4.8	23.8 ± 5.6	0.25
Stone size (cm), mean ± SD	4.2 ± 1.3	4.6 ± 1.5	0.18
Source of bacteria, *n* (%)		—	—
Mid-stream urine	118 (81.9)		
Pelvic urine	26 (18.1)		
Bacteria strains, *n* (%)			
*Escherichia coli*	—	50 (35)	—
*Enterococcus faecalis*		23 (16)	
*Klebsiella pneumoniae*		12 (8)	
*Streptococcus agalactiae*		10 (7)	
*Proteus mirabilis*		6 (7)	
*Others*		43 (27)	
Comborbidities, *n* (%)			0.22
Hypertension	28 (10)	17 (11.8)	
Diabetes mellitus	39 (14)	28 (19.4)	
History of stone surgery, *n* (%)			0.12
Open	8 (2.8)	5 (3.5)	
URL/RIRS	20 (7.2)	15 (10.4)	
PNL	18 (6.5)	10 (6.9)	
Preoperative serum creatinine (mg/dl), mean ± SD	1.2 ± 0.2	2.0 ± 0.7	0.02
Pre-operative management, *n* (%)			0.02
Double-J stent	4 (1.6)	10 (6.9)	
Nephrostomy	7 (2.4)	20 (13.1)	
Operative duration (min), mean ± SD	58.2 ± 17.2	69.2 ± 22.2	0.06
Number of access, mean ± SD	1.1 ± 0.2	1.7 ± 0.2	0.008
Hemoglobin loss (g/L), mean ± SD	1.6 ± 0.2	1.8 ± 0.3	0.18
1-month SFR, *n* (%)	249 (89.2)	108 (75)	0.02
Post-operative hospitalization (days), mean ± SD	5.6 ± 1.1	7.6 ± 2.1	0.03
Non-infectious postoperative complications, *n* (%)			1.0
Pleural injury	2 (0.72)	1 (0.69)	
Organ damage	/	/	
Embolization	2 (0.72)	1 (0.69)	
Stone composition, *n* (%)			0.03
Calcium-contained	225 (80.9)	80 (55.6)	
Struvite	26 (9.4)	37 (25.7)	
Uric acid	15 (5.4)	9 (6.3)	
Others	12 (4.3)	18 (12.5)	

### Surgical procedure

Total US-guided PNL was performed as previously described (12). A retrograde 5Fr ureteric catheter was first inserted into the renal pelvis with the patient in the lithotomy position. Then the patient was changed to prone position. A 3.5 MHz convex abdominal US transducer (Philips Healthcare, Eindhoven, The Netherlands) was used to detect the stones and collecting system. After successful puncture, Alken coaxial telescopic dilators (Richard Wolf GmbH, Knittlingen, Germany) or a high-pressure balloon dilator (X Force® N30 balloon dilator; Bard Urological, Covington, GA, USA) were used to establish the standard 24Fr tract. Stones were fragmented and suctioned with a combined ultrasonic/pneumatic lithotripter (Swiss LithoClast; Electro Medical Systems, Nyon, Switzerland) under the view of an 18Fr nephroscope in all patients. Irrigation flow speed was set at 400–500 ml/min in standard access. Additional access was made if necessary during the procedure, 24Fr was preferred but not being mandatory for the extra tracts. A 6Fr ureteral stent was placed routinely and kept indwelling for 2–4 weeks after surgery. A 14Fr nephrostomy tube was placed at the conclusion of surgery and removed 3–5 days later if no complications had occurred.

### Statistical analysis

Statistical analysis was performed using independent sample *t*-tests and *χ*^2^ tests. Medians ± standard deviation (SD) are reported for non-normally distributed continuous variables. Student's *t*-test was used to analyze continuous variables, whereas the *χ*^2^ or Fisher's exact test was used to analyze categorical variables. Statistical significance was set at a *p* < 0.05. Statistical analyses were performed using IBM SPSS version 21 (SPSS, Chicago, IL, USA).

## Results

The study cohort comprised 422 consecutive patients who had undergone PNL in our institution during the study period. The urine was sterile in 278 (65.9%) patients, whereas 144 (34.1%) patients had positive cultures (118 preoperative urine and 26 pelvic urine). *Escherichia coli* (*35%*), *Enterococcus faecalis* (*16%*), *Klebsiella pneumoniae* (*8%*), *Streptococcus agalactiae* (*7%*), *Proteus mirabilis* (*6%*) were the five most common bacteria in this series. Ten of the positive patients (7%) also had fungal infection (*Candida*) and 14 (9.7%) had multi-drug resistant organisms. Age and body mass index did not differ significantly between the two groups (*p* > 0.05). Stone size was also comparable in Groups 1 and 2 (4.2 ± 1.3 vs. 4.6 ± 1.5 cm; *p* = 0.18). The ratio of female patients was significantly higher in Group 2 (101; 71%) than in Group 1 (98, 35.3%; *p* < 0.001). The results of preoperative serum creatinine was much higher in Group 2 than Group 1 (2.0 ± 0.7 mg/dl vs. 1.2 ± 0.2 mg/dl; *p* = 0.02). In Group 2, 30 patients (20.8%) underwent pre-stent or nephrostomy because of severe urinary tract infection, whereas only 11 (4%) in Group 1underwent the above procedures. Ultrasound was used for guidance in all patients. The duration of surgery tended to be longer in Group 2 (69.2 ± 22.2 min) than in Group 1 (58.2 ± 17.2 min); however, this difference was not significant (*p* = 0.06). Fewer renal access were required in Group 1 than in Group 2 (1.1 ± 0.2 vs. 1.7 ± 0.2; *p* = 0.008). Duration of post-operative hospitalization was longer in Group 2 (7.6 ± 2.1 days) than in Group 1 (5.6 ± 1.1 d; *p* = 0.03). Post-operative KUB/CT was used to evaluate the residual stone status. The 1-month stone-free rate was significantly higher in Group 1 (249; 89.2%) than in Group 2 (108; 75%; *p* = 0.02). Stone composition was obtained in all patients. The proportion of infected stones in group 1 was significantly higher than that of negative group (*p* = 0.03). Relevant patient characteristics and perioperative data are listed in Table 1.

Infectious complications were classified into four grades in accordance with the Clavien–Dindo system. The rate of Grade 1 complications (fever >38°C) was much higher in the positive than in the group 2 (*p* = 0.04); however, the rate of severe complications did not differ significantly between the groups (Grades II, III, IV). The two groups had similar sepsis rates. None of our study patients developed septic shock or required transfer to the intensive care unit. The details of post-operative infectious complications are shown in Table 2.

**Table 2:** Post-operative infectious complications according to clavien-dindo grade classification.

Grade	Group 1	Group 2	*P* value
I (fever >38 °C), *n* (%)	21 (7.6)	22 (15.3)	0.04
II (Urosepsis needs additional drug intervention), *n* (%)	13 (4.7)	10 (6.9)	0.09
III (Urosepsis needs surgical intervention), *n* (%)	/	/	/
IV (septic shock), *n* (%)	/	/	/

## Discussion

Percutaneous nephrolithotomy is the first-line treatment for complex kidney stones. Infectious complications may be life-threatening and require close attention. Several risk factors for fever and sepsis have been identified, including presence of urinary infection, struvite stones, long duration of surgery, and large stone burden. Positive preoperative urine cultures are an independent risk factor for postoperative fever and sepsis. According to available guidelines and expert consensus, a negative preoperative urine culture is necessary for PNL (16). However, under the high hydrostatic pressure generated by injecting the irrigation fluid, stone-colonizing bacteria may continue to be released and immigrate into blood vessels until the fragmenting procedure has been completed. Additionally, even with long term anti-inflammatory or drainage therapy, it is difficult to achieve sterile urine in patients with stones and pyonephrosis (17). Furthermore, previous studies have found that postoperative infections may occur in patients with negative preoperative urine cultures, indicating that urine culture status may not play a key role in predicting development of postoperative infection.

Infectious complications in patients with negative preoperative cultures have been reported by several groups of researchers. Chen et al. found that more than 50% of patients who developed urosepsis after PNL had sterile urine preoperatively (18). Singh et al. reported a 1.09% incidence of septic shock in patients with negative preoperative urine cultures (19). Possible explanations include the following. First, the results of mid-stream urine cultures do not reliably indicate the overall status of the urinary tract. Incarcerated stones can completely obstruct the urinary tract, preventing discharge of bacteria-laden urine. Second, bacteria within stones can lead to false negative urine samples. Endotoxins released into the systematic circulation can result in postoperative fever and sepsis. De Lorenzis et al. found that pathogens colonizing stones differ from those isolated from urine and recommended that stone or fragment cultures should be performed to more accurately identify infections (20). In contrast, Osman et al. stated that stone cultures should not be routinely implemented because in most cases the regimen need not be changed (21). We do not routinely perform stone cultures in our unit because we used to find that the bacteria in stones were consistent with those in urine. Thus, in our experience, stone culture results do not provide necessary guidance on postoperative anti-infection regimens.

In addition to preoperative risk factors, IPP also plays a key role in predicting postoperative infections after endourological procedures. It has been well demonstrated that high IPP is the driving force behind bacteria entering the circulatory system through interlobar veins. The threshold pressure for retrograde flow into veins has been estimated to be 30 mmHg. During PNL, there is a dynamic environment of fluid flux. More recent studies have reported an association between use of higher volumes of irrigation fluid and increased risk of infections (22). In addition to the irrigation factor, size and number of accesses also affect the IPP. Ablourbih et al. found that, when using a rigid nephroscope, IPP was higher when there was one rather than two tracts (31.35 mmHg vs. 9.35 mmHg; *p* < 0.001) (23). Loftus et al. found that IPP was higher in a mini-PNL group (18.76 ± 5.82 mmHg) than in a standard tract group (13.56 ± 5.82 mmHg) and that more time was spent above 30 mmHg in patients in the mini-PNL arm (24). Lithotripsy devices with active suction function are an important tool for reducing IPP during PNL. Ablourbih et al. also found that the use of suction resulted in a significantly lower pressure in patients with one tract (−1.3 mmHg) than in those with two (1.8 mmHg) (23). In our series, the urine culture group 1 had significantly more accesses than did the group 2 (1.7 ± 0.2 vs. 1.2 ± 0.2, *p* = 0.008). In the present study, IPP was not routinely tested. IPP was monitored in a limited number of patients and was shown to be below 30 mmHg throughout the whole procedure, the irrigation speed was commonly set at 400–500 ml/min, which promotes both a clear field of vision and a relatively low IPP. Furthermore, we routinely administered dexamethasone and furosemide during surgery in both groups, recent studies having shown that these drugs reduce postoperative inflammatory reactions, resulting in better clinical outcomes (25). This may explain the lower overall rate of postoperative infection in the enrolled patients compared with that in previous studies. According to previous studies, duration of intrapelvic pressure over 30 mmHg also has an important impact on postoperative fever. We did not monitor IPP continuously because of equipment problems.

*E. coli* was the most commonly isolated micro-organism in the present study, accounting for 35% of positive cultures. This finding is consistent with prior studies of patients with urinary tract infection, in which *E. coli* was the predominant bacterium in the bladder (2). All 278 patients were given prophylactic antibiotics (second generation cephalosporin or levofloxacin) for 1–3 days. Broad-spectrum antibiotics were administered intravenously for 2–7 days before surgery to patients with positive cultures. Those with bacteria in their urine one day before surgery were classified into the group 1. Ten patients (7%) also had fungal infections and 14 (9.7%) were infected with multi-drug resistant organisms. The proportion of patients with complex urinary tract infections was higher than in many previous studies. This may be attributable to the fact that we are the major urolithiasis prevention and treatment base in the north of our country. Thus, numerous patients with refractory stones, many of whom have undergone multiple anti-inflammatory and surgical treatments in their local hospitals, are referred to us every year. A considerable proportion of our patients needs stenting or nephrostomy to control systemic or focal infection prior to lithotomy.

In our study, the ratio of female patients tended to be higher in the positive than in the group 2; this is consistent with published studies that found that female patients more readily develop urinary infections than do male patients (26). The stone burdens were similar in the positive and group 2s. We speculate that this may have been attributable to the higher incidence of infected calculi in the group 1. Stones can grow more quickly in infected urine. The residual stone rate was higher in the sterile than the group 1, possibly because the presence of infection concerns surgeons to the degree that they perform the procedure as quickly as possible. In addition, infective foci and small stones are sometimes difficult to detect accurately during US-guided surgery.

Although prediction of postoperative infection on the basis of preoperative urine culture is not completely reliable, in our study we found that patients with positive bladder cultures and pyuria had a higher rate of postoperative fever. However, there was no difference between the groups in the rate of serious complications (sepsis and shock). In this study, systemic inflammatory-response syndrome (SIRS) was not used as an indicator of postoperative infection because we believe that SIRS has high sensitivity and low specificity for identifying sepsis. Many patients with fever meet the diagnostic criteria for SIRS. Another indicator that been widely used recently, rapid sepsis-related organ failure assessment, may more accurately indicate sepsis status. Furthermore, the cut-off used to define fever varies between studies. Our threshold was 38 °C rather than 38.5°C; this may have influenced our findings. Indeed, in one published study the proportion of PNL patients who developed a post-operative fever of >38.5°C was 16.7%, whereas 28.8% of patients had temperatures >38°C (2).

Our study focused on the feasibility and safety of PNL in patients with poor preoperative control of infection. We found comparable overall complication rates in the two groups. The stone-free rate was acceptable compared with rates in previous studies. Nevertheless, our study had several limitations. First, it was retrospective and therefore inherently subject to bias; prospective randomized studies are required to further investigate our findings. Second, postoperative fever and sepsis were defined as occurring within 24 h after surgery. Subsequent temperatures were not considered despite a minority of patients having delayed fever. Third, several patients did not undergo CT scans to assess residual stones, which may have increased the rate of false negatives. Last but not least, we did not perform stone cultures in our study; this may also have contributed to our false negative rate. About half of patients with negative bladder urine cultures reportedly have positive stone cultures (11).

## Conclusions

In this study, we found that PNL performed under certain conditions is safe and feasible in patients with infected urine. Though postoperative fever occurred more frequently in patients with positive cultures, sepsis and septic shock rates were comparable to those in the negative urine group. Negative urine culture is not necessary for ultrasound guided PNL under certain condition. Knowledge of the factors that influence intra-pelvic pressure and methods of controlling it may play a more important role in improving patient safety during PNL.

## Data Availability

The original contributions presented in the study are included in the article/Supplementary Material, further inquiries can be directed to the corresponding author.
